# Randomized, Ascending Dose, Phase 2 Study of KHK4083, an Anti-OX40 Monoclonal Antibody, in Moderately Active Ulcerative Colitis

**DOI:** 10.1093/crocol/otaa049

**Published:** 2020-06-09

**Authors:** Jaroslaw Kierkus, Marina Pesegova, Maria Klopocka, Marija Brankovic, Noriyuki Kasai, Sergey Efuni, Jennifer Kong, Yu Nakajima, Christina Jordan, Takeshi Matsui, Brian G Feagan, Vincent Strout

**Affiliations:** 1 Maternal, Pediatric and Adolescent Healthcare Centre, Gastroenterology Diagnostic Facility for Adults, Warsaw, Poland; 2 Department of Gastroenterology, Territorial Clinical Hospital, Krasnoyarsk, Russia; 3 Department of Gastroenterology and Nutrition, Nicolaus Copernicus University in Toruń, Collegium Medicum in Bydgoszcz, Poland; 4 Gastroenterology Clinic, Bezanijska Kosa Clinical Hospital Centre, Belgrade, Serbia; 5 Kyowa Kirin Pharmaceutical Development, Inc., Princeton, New Jersey; 6 Robarts Clinical Trials, Western University, London, Ontario, Canada

**Keywords:** clinical study, KHK4083, OX40, ulcerative colitis

## Abstract

**Background:**

OX40 (CD134) plays a role in the maintenance of late T-cell proliferation and survival. KHK4083 is a monoclonal antibody directed against OX40. We aimed to assess the safety and preliminary efficacy of KHK4083 in patients with moderately active ulcerative colitis (UC).

**Methods:**

In this multicenter, double-blind, parallel-group, phase 2 study, patients with moderately active UC patients were randomized to ascending doses of intravenous KHK4083 (1, 3, or 10 mg/kg) or placebo every 2 weeks for 12 weeks. The primary endpoint was safety. The primary efficacy end point was the change from baseline in mean modified Mayo endoscopy subscore at week 12. Treatment with KHK4083 or placebo was continued every 4 weeks for up to 52 weeks in responders.

**Results:**

Long-term treatment with KHK4083 was well tolerated, with treatment-related adverse events being predominantly transient mild-to-moderate infusion-related reactions. Exploratory analysis of biopsy samples showed the virtually complete elimination of OX40+ cells in colon mucosa after 12 weeks of KHK4083 treatment. There were no significant differences between any of the randomized KHK4083 dose groups and placebo for the mean change in Mayo endoscopy subscore from baseline to week 12.

**Conclusions:**

KHK4083 can be safely administered intravenously at doses up to 10 mg/kg every 2 or 4 weeks for up to 52 weeks. Proof of pharmacodynamic action was confirmed by depletion of the elevated levels of the OX40+ cells associated with UC at all tested doses. Clinical response and mucosal healing (endoscopic improvement) in this population was not correlated with ablation of OX40+ T cells.

## INTRODUCTION

Ulcerative colitis (UC) is a chronic, relapsing/remitting, inflammatory disorder of the colonic mucosa.^1–3^ Pathologically, UC is characterized by the presence of increased numbers of natural killer T cells and an atypical interleukin (IL)13-induced T-helper 2 immune response.^4^ OX40 (cluster of differentiation 134, tumor necrosis factor [TNF] receptor super family 4) offers a potential novel therapeutic target in UC (Supplementary Fig. 1).^5^ OX40, which has a role in the maintenance of late T-cell proliferation and survival by apoptosis suppression and memory T-cell induction,^5–10^ is overexpressed on lamina propria T cells from patients with UC.^11–13^ Furthermore, treatment of mice with either trinitrobenzene sulfonic acid-induced or IL2 knockout colitis with an OX40-immunoglobulin fusion protein inhibited mucosal inflammation.^14^

KHK4083 is a fully human, nonfucosylated IgG_1_ monoclonal antibody directed against OX40, that demonstrates antagonistic activity to OX40 ligand in vitro and efficacy in animal models of graft-versus-host disease and delayed-type hypersensitivity (unpublished data on file, Kyowa Kirin Pharmaceutical Development, Inc.). KHK4083 may therefore have potential in the treatment of UC patients. Single doses of intravenous (IV) KHK4083 of up to 10 mg/kg have been shown to be well tolerated in a phase 1 trial of patients with plaque psoriasis.^5^

The aim of this trial was to evaluate the safety and efficacy of IV administered KHK4083 in patients with moderately active UC who had been previously unsuccessfully treated with corticosteroids, immunosuppressants, or TNFα antagonists.

## METHODS

The study protocol and its subsequent amendments were approved by the relevant Institutional Review Board/Independent Ethics Committee at the participating study centers. The study was conducted in accordance with the Declaration of Helsinki, International Conference on Harmonization of Good Clinical Practice guidelines, and any applicable national laws and regulations, and is registered at ClinicalTrials.gov (NCT0264786). All patients provided written, informed consent prior to participation.

### Patients

Male or female patients ≥18 years of age with moderately active UC (total Mayo score 4–9, modified Mayo endoscopy subscore (mMES) ≥ 2 and extending ≥15 cm from the anal verge) with a duration of UC diagnosis >6 months were eligible for inclusion. They had to have received treatment with corticosteroids, immunosuppressants, or TNFα antagonists in the previous 5 years that had been unsuccessful because of lack of efficacy or intolerance. Concurrent oral UC therapy was permitted with stable doses of corticosteroids (prednisolone ≤20 mg/d or equivalent, or budesonide ≤9 mg/d) for ≥2 weeks (or ≥4 weeks if started <8 weeks) and aminosalicylates (mesalazine ≤4.8 g/d or sulfasalazine ≤3 mg/d) for ≥2 weeks, and/or azathioprine ≤3 mg/kg/d or 6-mercaptopurine ≤1.5 mg/kg/d for ≥12 weeks before screening.

Major exclusion criteria were as follows: failure of UC to respond to ≥2 biologic agents with different mechanisms of action (e.g., infliximab, vedolizumab) or ≥3 TNFα biologics (e.g., inflixumab, adalimumab, golimumab); and prior lymphocyte-depleting therapy (e.g., natalizumab, efalizumab, rituximab, cyclophosphamide, chlorambucil, total lymphoid irradiation) at any time; and use of TNFα antagonists ≤8 weeks (or 5 half-lives not exceeding 12 weeks), vedolizumab ≤16 weeks, immunotherapies (e.g., methotrexate, cyclosporine, mycophenolate, tacrolimus) ≤4 weeks, 5-ASA enema or steroid enema or suppository ≤2 weeks, and investigational agents ≤8 weeks (or 5 half-lives) before randomization. Other exclusion criteria included non-UC gastrointestinal disease; previous or anticipated UC surgery; and a history of clinically significant cardiac, renal, pulmonary, immunological, or autoimmune disease apart from UC. Detailed information of inclusion and exclusion criteria is provided as Supplementary Material.

### Study Design

**Figure 1.**
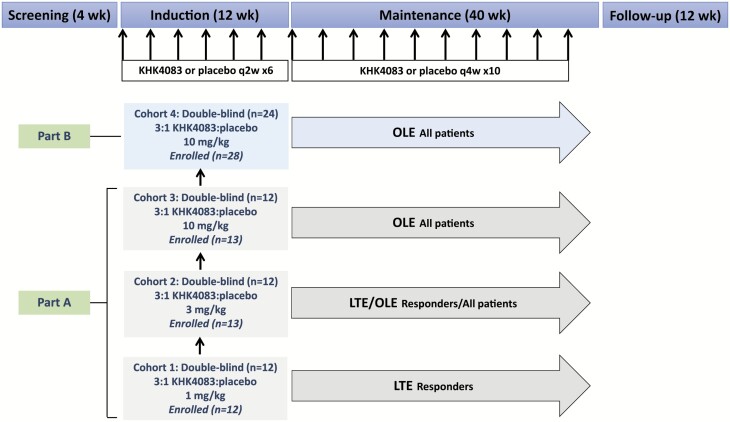
Study plan showing numbers of patients planned for enrollment and actually enrolled. LTE, long-term double-blind extension; OLE, open-label extension.

The design of this multicenter study is outlined in Figure 1. The initial randomized, 12-week, double-blind, induction phase comprised parts A and B. Ascending doses of KHK4083 (1, 3, and 10 mg/kg in cohorts 1, 2 and 3, respectively, with 12 patients planned for each cohort) were evaluated in part A. The KHK4083 dose recommended following safety review in part A was evaluated in part B (10 mg/kg in dose-expansion cohort 4, with 24 patients planned). In each cohort, patients were randomized in a 3:1 ratio to KHK4083 or placebo administered every 2 weeks. This initial phase was followed by a 40-week maintenance phase during which KHK4083 or placebo doses were administered every 4 weeks. In the original protocol, clinical responders could elect to continue maintenance treatment (KHK4083 or placebo) during a long-term extension under continued double-blind conditions. This was changed by protocol amendment during recruitment into cohort 2. Protocol amendments are described in Supplementary Material. Subsequently, all patients regardless of response could elect to receive maintenance KHK4083 (at the dose level randomized during induction irrespective of whether they had received KHK4083 or placebo) during open-label extension. Patients participating in the double-blind long-term extension phase who experienced clinical worsening or disease flare on placebo were allowed to transition to the open-label extension after switching to KHK4083 at the dose level of the original cohort.

KHK4083 and placebo were administered by IV infusion over 60 min. Randomization was performed using a computer-generated scheme. Concurrent UC medication including corticosteroids could be tapered at the discretion of the investigator during the long-term maintenance phase. Rescue treatment with systemic or oral corticosteroids was permitted during the long-term maintenance phase.

The dose selection in this study was based on considerations of safety margins and the predicted pharmacokinetics (PK) profiles at the intended doses (for details see Supplementary Material).

### Objectives

The primary objectives were to determine the safety and tolerability of administration of multiple ascending doses of KHK4083 in part A to select the highest dose tolerated by patients with moderately active UC to recommend for use in part B and, in part B, to determine whether the recommended dose of KHK4083 improved endoscopic appearance at week 12. The assessment was based on a mean change from baseline to week 12 in mMES. Secondary objectives included additional analyses of efficacy, safety, PK, and immunogenicity. Exploratory objectives included further analyses of efficacy, pharmacodynamics (PD) [biomarkers], and PK–PD relationships. Secondary and exploratory analyses are detailed in Supplementary Material.

### Safety Assessments

Adverse events (AEs) were recorded following observation or in response to nonleading questioning by the investigator at clinical visits or following spontaneous reporting by the patient at any time. Safety was determined from AEs, physical examination findings, electrocardiogram readings, vital sign measurements, and clinical laboratory test results (serum chemistry, hematology, coagulation profile, and urinalysis) recorded throughout treatment and during the 12-week follow-up period. AEs were graded for intensity (mild, moderate, or severe) and relationship to treatment (related or unrelated), and coded according to Medical Dictionary for Regulatory Activities, version 18.1. Serious AEs (SAEs) were reported in an expedited manner. Immunogenicity was determined using a validated electrochemiluminescent (ECL) ligand-binding assay for the detection of anti-KHK4083 antibodies in serum. The safety analysis set included all patients who received study medication.

### Efficacy Assessments

The efficacy variables included mMES (endoscopic subscores from 0 to 3 from the total Mayo score^15^ with modification of the endoscopy score by excluding mild friability from the subscore of 1 as recommended by the US Food and Drug Administration^16^), Ulcerative Colitis Endoscopic Index of Severity (UCEIS) score, modified Baron endoscopic score, total Mayo score, and partial Mayo score (without sigmoidoscopy) measured at baseline, week 12, and week 52. The primary efficacy end point was the change in mMES from baseline to week 12 in the randomized groups receiving KHK4083 (1, 3, or 10 mg/kg) compared with placebo. Clinical response was defined as a total Mayo score improvement by ≥3 points and by ≥30% from baseline, and a decrease of ≥1 on the rectal bleeding subscale from baseline or an absolute rectal bleeding subscore of 0 or 1. Clinical remission was defined as a total Mayo score of ≤2 and no subscore >1. Endoscopy scoring was determined by a central reader. Mucosal healing (endoscopic improvement) was defined by an mMES of 0 or 1. For histological mucosal healing assessment, Robarts Histopathology Index (RHI) scoring was performed using hematoxylin/eosin (H&E)-stained slides.^17^ Total RHI score ranges from 0 (no disease activity) to 33 (severe disease activity). The RHI was scored by a single gastrointestinal histopathologist blinded to the patients’ clinical information and endoscopic findings. A single pathologist was used to score histology to minimize variability due to interobserver variances.

### Pharmacokinetic and Immunogenicity Assessments

Blood samples were collected at specified time points for the duration of the study for determination of serum KHK4083 concentrations using a validated ECL immunoassay (Syneos Health, Princeton, NJ). The dynamic range of the assay was 25–10,000 ng/mL. Pharmacokinetic parameters were determined by non-compartmental analysis at weeks 0, 10, and 48 in cohorts 1–3 using a validated SAS macro (version 9.3, SAS Institute Inc., Cary, NC). The detection of anti-KHK4083 antibodies in human serum samples was performed using a validated ECL assay. A tiered approach (screening assay, confirmatory assay, and titer determination assay) was utilized for clinical sample analysis. The drug tolerance limit was 100 µg/mL.

### Biomarker Assessments

OX40+ cells in rectal biopsies were analyzed by immunohistochemistry using sigmoidoscopy biopsy specimens. Biopsy samples (n = 2–4) were fixed in phosphate-buffered formalin, embedded in paraffin and cut into 4-µm slices. Immunohistochemistry staining of OX40 was performed using Autostainer Link 48 (Dako North America, Inc.) with a primary monoclonal antibody against human OX40 which is noncompeting with KHK4083 (ACT-35, ThermoFisher Scientific, Inc.), a secondary antibody (EnVision + System/HRP, mouse; Dako North America, Inc.), and a detection kit (Liquid DAB + Substrate ChromogenSystem, Dako North America, Inc.). Whole-slide scanning of stained slices was performed using a Brightfield slide scanner (Leica Microsystems K.K.) at 20× magnification. Image processing software (ImageScope and Aperio ePathology Solutions, Aperio Technologies, Inc.) was used for counting the number of OX40+ cells. Mucosa was selected for analysis areas based on the images of each H&E-stained slide. The staining intensity was classified into 4 levels (0, 1+, 2+, 3+) and the number of cells with not less that 1+ staining intensity was recognized as OX40-positive cells. Representative images of OX40-stained cells from a patient at baseline and after 12 weeks of KHK4083 treatment are shown in Supplementary Figure 2. Areas that might affect analysis (e.g., wrinkles, peeling, nonspecific staining areas) were excluded. Regions of underlying muscularis mucosa and submucosa with associated tissue areas were also excluded from analysis. Staining intensity was classified as 4 levels (0+, 1+, 2+, and 3+). The number of OX+ cells (classed as 1+ or above) and total analysis area (mm^2^) were calculated.

Serum levels of 31 cytokines and chemokines were measured using the ECL Meso Scale Discovery 31-plex assay method: IL1β, IL2, IL4, IL12p70, IL13, IL10, IL6, IL8, TNFα, interferon-γ, granulocyte-macrophage colony-stimulating factor, IL5, IL17A, TNFβ, IL7, IL1α, IL15, IL12/IL23p40, IL16, eotaxin, eotaxin-3, macrophage inflammatory protein 1α, macrophage inflammatory protein 1β, thymus- and activation-regulated chemokine, interferon-γ-inducible protein-10, monocyte chemoattractant protein 1, macrophage-derived chemokine, monocyte chemoattractant protein 4, vascular endothelial growth factor (VEGF)-A, VEGF-C, and VEGF-D.

### Sample Size

In cohorts 1–3, the sample size (n = 12 per cohort with 3:1 ratio for KHK4083:placebo) was based on the feasibility of the safety and tolerability assessment to recommend starting the treatment of the next cohort. In cohort 4, the sample size (*n* = 24 with 3:1 ratio for KHK4083:placebo) was based on the feasibility of exploratory assessments in efficacy and safety at the recommended dose level. Based on the UCEIS and Mayo scores and from combining cohorts 1–4, the plan was to have a total of approximately 27 randomized patients receive the recommended dose of KHK4083 and 15 patients receive placebo. Assuming a 15% rate of unevaluable patients, it was anticipated that approximately 60 patients were required to be randomized in order to achieve 51 evaluable patients in the full analysis set.

### Statistical Analysis

Categorical data were summarized by calculating the number and percentage, whereas continuous variables were summarized by calculating descriptive statistics (number of patients, mean, SD, and minimum, median, and maximum values).

The primary end point was analyzed using an analysis of covariance (ANCOVA) model in the full analysis set. Treatment was considered as the fixed effect and baseline mMES was considered as covariates in the model. The adjusted least-squares (LS) mean and its 95% confidence interval were calculated for each treatment dose group from the ANCOVA model. No adjustment for multiplicity was planned for this study. Difference in LS mean from placebo for each treatment dose was calculated with the relative 95% confidence interval and *P* value. Similarly, UCEIS and total Mayo Clinic score were analyzed using ANCOVA in the full analysis set with their baseline values as covariate. For further investigation of the data, clinical response, clinical remission, and mucosal healing at week 12 were evaluated in the intent-to-treat population, which was not a prespecified population in protocol. In addition, as post hoc analyses, clinical status at weeks 12 and 52 was evaluated for those patients who completed endoscopic assessments at both of the time points. SAS 9.4 was used for preceding analyses. All the variables of the biomarker data were tabulated and statistically analyzed using JMP 14.0 software (JMP, Cary, NC). Tukey’s test was used post hoc to compare KHK4083- and placebo-treated groups and to compare nonresponder and responder (response/remission) groups, with nominal *P* values < 0.05 being considered of interest.

## RESULTS

### Study Population

The study was conducted from June 2016 to October 2018. The safety analysis set (all 66 randomized patients) were recruited at 23 centers in 7 countries (United States, Poland, Hungary, Czech Republic, Romania, Serbia, and Russia). The CONSORT diagram for the disposition of the patients during the 12-week randomized induction phase is shown in Supplementary Figure 3. Of 66 patients randomized, the full analysis set included all patients (n = 52, 78.8%) who received at least 1 full dose of study treatment (KHK4083 or placebo) and had a baseline and at least 1 post-treatment primary efficacy variable. Throughout the study (including long-term treatment), 28 patients (42.4%) discontinued for the following reasons: AEs (n = 5), disease worsening (n = 5), consent withdrawal (n = 12), investigator discretion (n = 4), death (n = 1), and other reason (n = 1).

The demographic and baseline clinical characteristics of the patients including prior UC medication use are summarized in Supplementary Table 1. Demographics were generally similar across the cohorts, although there may have been a degree of imbalance between the patients who received KHK4083 and placebo. Previous use of immunosuppressants, TNFα antagonists, and 5-aminosalicylate formulations appeared more frequent in the placebo recipients. Approximately half of the patients overall had previously received systemic corticosteroids.

### Safety

AEs are summarized in Table 1. There was no apparent dose-related increase in treatment-emergent or treatment-related AEs in patients receiving KHK4083. The most common treatment-emergent AEs (occurring in ≥5% of patients) across all KHK4083 doses were anemia (12.2%), pyrexia (12.2%), UC (10.2%), and arthralgia (6.1%). Treatment-related AEs occurring in ≥1 patient across all doses were pyrexia (n = 4, 8.2%) and chills (n = 3, 6.1%). Seven of 66 (10.6%) patients experienced mild (n = 5) or moderate (n = 2) infusion-related reactions, all in patients receiving KHK4083 during the double-blind induction phase. KHK4083 was discontinued for a treatment-related AE in one patient (mild infusion-related reaction).

**Table 1.** Adverse Events (Safety Analysis Set)

	No. of patients (%)					
	1 mg/kg (n = 9)	3 mg/kg (n = 10)	10 mg/kg (n = 30)	All doses (n = 49)	Placebo (n = 17)*	All patients (n = 66)
Any treatment-emergent AE	5 (55.6)	7 (70.0)	21 (70.0)	33 (67.3)	13 (76.5)	46 (69.7)
Any treatment-related AE	3 (33.3)	2 (20.0)	6 (20.0)	11 (22.4)	2 (11.8)	13 (19.7)
Any treatment-emergent SAE	1 (11.1)	1 (10.0)	4 (13.3)	6 (12.2)	3 (17.6)	9 (13.6)
Any treatment-related SAE	0	0	0	0	0	0
Death	0	1 (10.0)	0	1 (2.0)	0	1 (1.5)
Any discontinuation for treatment- emergent AE	0	1 (10.0)	3 (10.0)	4 (8.2)	1 (5.9)	5 (7.6)
Any discontinuation for treatment- related AE	0	0	1 (3.3)	1 (2.0)	0	1 (1.5)
Most common^†^ treatment-emergent AEs by preferred term^‡^						
Colitis ulcerative	1 (11.1)	1 (10.0)	3 (10.0)	5 (10.2)	5 (29.4)	10 (15.2)
Anemia	1 (11.1)	2 (20.0)	3 (10.0)	6 (12.2)	3 (17.6)	9 (13.6)
Pyrexia	3 (33.3)	1 (10.0)	2 (6.7)	6 (12.2)	0	6 (9.1)
Arthralgia	2 (22.2)	0	1 (3.3)	3 (6.1)	1 (5.9)	4 (6.1)
Most common^¶^ treatment-related AEs by preferred term^‡^						
Pyrexia	2 (22.2)	1 (10.0)	1 (3.3)	4 (8.2)	0	4 (6.1)
Chills	1 (11.1)	1 (10.0)	1 (3.3)	3 (6.1)	0	3 (5.5)

MedDRA, Medical Dictionary for Regulatory Activities.

*Includes patients who changed from placebo to KHK4083 in the open-label extension period.

^†^Occurring in ≥5% of patients overall.

^‡^Coded by MedDRA v18.1.

^¶^Occurring in ≥1 patient overall.

There were no treatment-related SAEs or deaths. Treatment-emergent SAEs occurred in 8 patients receiving KHK4083: UC (n = 2), and UC+pneumonia+blood albumin decreased, abscess drainage, myocardial infarction (MI), anemia, post-procedural infection (eye infection following elective bilateral laser vision correction), and *Clostridium difficile* infection (each n = 1). One patient died from MI during the study, which was considered unrelated to study treatment by the investigator. The patient was a 59-year-old male with a history of MI and hypertension approximately 2.5 years prior to trial entry, and mitral insufficiency was recorded in his medical history. The patient initially experienced grade 2 MI as an SAE on day 76 while receiving KHK4083 3 mg/kg every 2 weeks. He was successfully treated with balloon dilatation and discharged on day 83. Approximately 4.5 months later while receiving KHK4083 3 mg/kg every 4 weeks, he died suddenly from MI when retiring to bed at home.

### Efficacy

The mean change in mMES from baseline to week 12, the primary efficacy end point, is shown in Table 2. Similar decreases in mMES from baseline to week 12 with KHK4083 (3 and 10 mg/kg) and placebo were observed. There was no statistically significant difference for the LS mean change from baseline to week 12 in mMES for any dose of KHK4083 compared with placebo. Secondary endpoints also showed no significant differences for changes in efficacy parameters from baseline to week 12 for any dose of KHK4083 compared with placebo (results for UCEIS and total Mayo score are shown in Supplementary Tables 2 and 3, respectively). Clinical response, clinical remission, and mucosal healing rates at week 12 for the intent-to-treat population as a post hoc analysis did not reveal any differences between any dose of KHK4083 and placebo (Supplementary Table 4).

**Table 2.** mMES: Actual and Change from Baseline at Week 12 (Full Analysis Set)

	KHK4083 Dose				Placebo (n = 15)
	1 mg/kg (n = 7)	3 mg/kg (n = 8)	10 mg/kg (n = 22)	All doses (n = 37)	
Baseline					
Mean (SD)	3.0 (0.0)	2.8 (0.5)	2.7 (0.5)	2.8 (0.4)	2.6 (0.5)
Week 12					
Mean (SD)	2.6 (0.8)	1.9 (1.1)	2.1 (1.1)	2.2 (1.1)	2.1 (1.0)
Change from baseline to week 12					
Mean (SD)	–0.4 (0.8)	–0.9 (1.0)	–0.5 (1.3)	–0.6 (1.1)	–0.5 (0.8)
95% CI	–1.2 to 0.3	–1.7 to 0.0	–1.1 to 0.0	–1.0 to –0.2	–1.0 to –0.1
LSM (SE)	–0.260 (0.403)	–0.853 (0.366)	–0.563 (0.221)	—	–0.599 (0.270)
95% CI of change	–1.070 to 0.550	–1.590 to –0.116	–1.007 to –0.118	—	–1.141 to –0.056
Difference in LSM (SE) versus placebo*	0.338 (0.492)	–0.254 (0.456)	0.036 (0.348)	—	—
95% CI of difference	–0.651 to 1.328	–1.172 to 0.663	–0.664 to 0.735	—	—
*P* value	0.495	0.580	0.919	—	—

CI, confidence interval; LSM, least-squares mean;

*Comparison between treatments (each KHK4083 dose group vs. placebo) was performed using an analysis of covariance model with change from baseline as the dependent variable and treatment group as the fixed effect, and baseline value as covariate.

The efficacy analysis at week 52, the end of the maintenance phase, was complicated by the limited number of patients completing treatment and undergoing sigmoidoscopy. Thirty-six patients completed endoscopic assessment at week 52, which included 27 patients who had continuously received KHK4083 from baseline and 9 patients who initially received placebo and were switched to KHK4083 at or after week 12. Post hoc evaluation of the rates of clinical response, clinical remission, and mucosal healing were not significantly different from week 12 after the additional 36 weeks of treatment with KHK4083 for those patients who completed both week 12 and 52 assessments (Supplementary Table 5). Of the 27 patients initially randomized to KHK4083 at baseline who completed week 52, 5 (18.5%) had mucosal healing at week 12 but not at week 52, 6 (22.2%) had the opposite outcome, 5 (18.5%) had mucosal healing at weeks 12 and 52, and 11 (40.7%) had no mucosal healing at weeks 12 and 52. Corresponding results for the 9 patients who initially received placebo and completed week 52 were 2 (22.2%), 2 (22.2%), 1 (11.1%), and 4 (44.4%), respectively.

**Figure 2.**
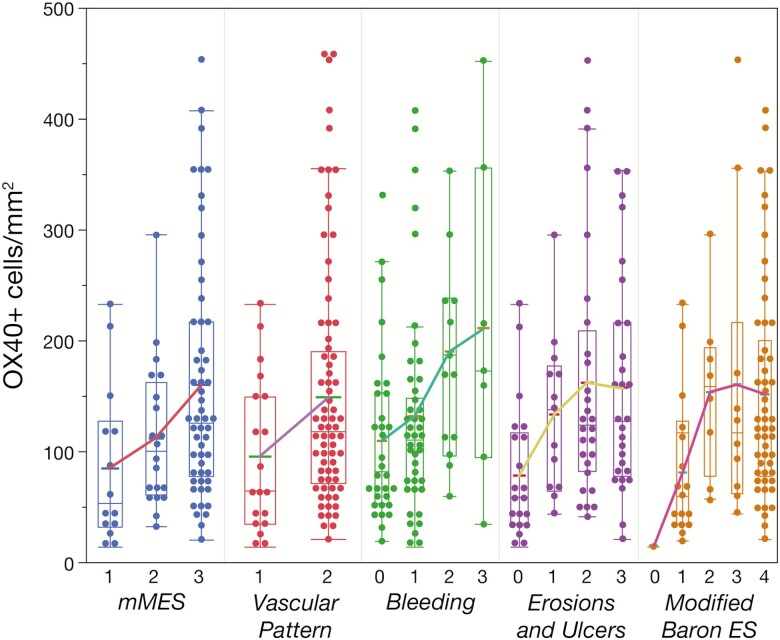
Box plots of correlation between OX40+ cell density and endoscopic scores at baseline for 90 patients (66 initially enrolled in the double-blind study and 24 not enrolled at screening). Each point represents the mean of multiple (up to 4) tissue biopsy specimens for each patient. The lines connect means of the box plots. ES, endoscopy score.

Notwithstanding the clinical findings, there was positive trend in the correlation between OX40+ cell density and endoscopic scores, which suggests OX40+ cell density is associated with severity of inflammation (Fig. 2). Correlation between baseline RHI and clinical response at week 12 is shown in Supplementary Figure 4. In the placebo-treated group, most patients had an RHI lower than the median (i.e., less severe disease), and in the KHK4083-treated group (all doses combined), a higher proportion of patients had higher RHI (i.e., more severe disease), which indicates a degree of imbalance in RHI score at baseline. With less severe disease at baseline, the response rate at week 12 was high regardless of treatment (65% on KHK4083 and 78% on placebo). With more severe disease at baseline, the response rate at week 12 was higher with KHK4083 than on placebo (60% vs. 33%). With less severe disease, the mucosal healing rate was higher with KHK4083 than on placebo (53% vs. 33%). With more severe disease, the mucosal healing was similar with KHK4083 and placebo (15% vs. 17%), although the numbers evaluable were low. The mucosal healing rate for the KHK4083-treated group was much lower with more severe disease than with less severe disease (15% vs. 53%).

Wide-range C-reactive protein and fecal calprotectin were measured longitudinally but, due to high interindividual variability, no conclusion could be drawn regarding these evaluations and they are not reported. Inflammatory Bowel Disease Questionnaires were completed longitudinally to evaluate the activity of KHK4083 on health-related quality of life, but no improvement was detected over time versus baseline or compared with placebo.

### Pharmacokinetics and Immunogenicity

These data are presented in Supplementary Results, Supplementary Table 6, and Supplementary Figure 5.

### Biomarker Analysis

**Figure 3.**
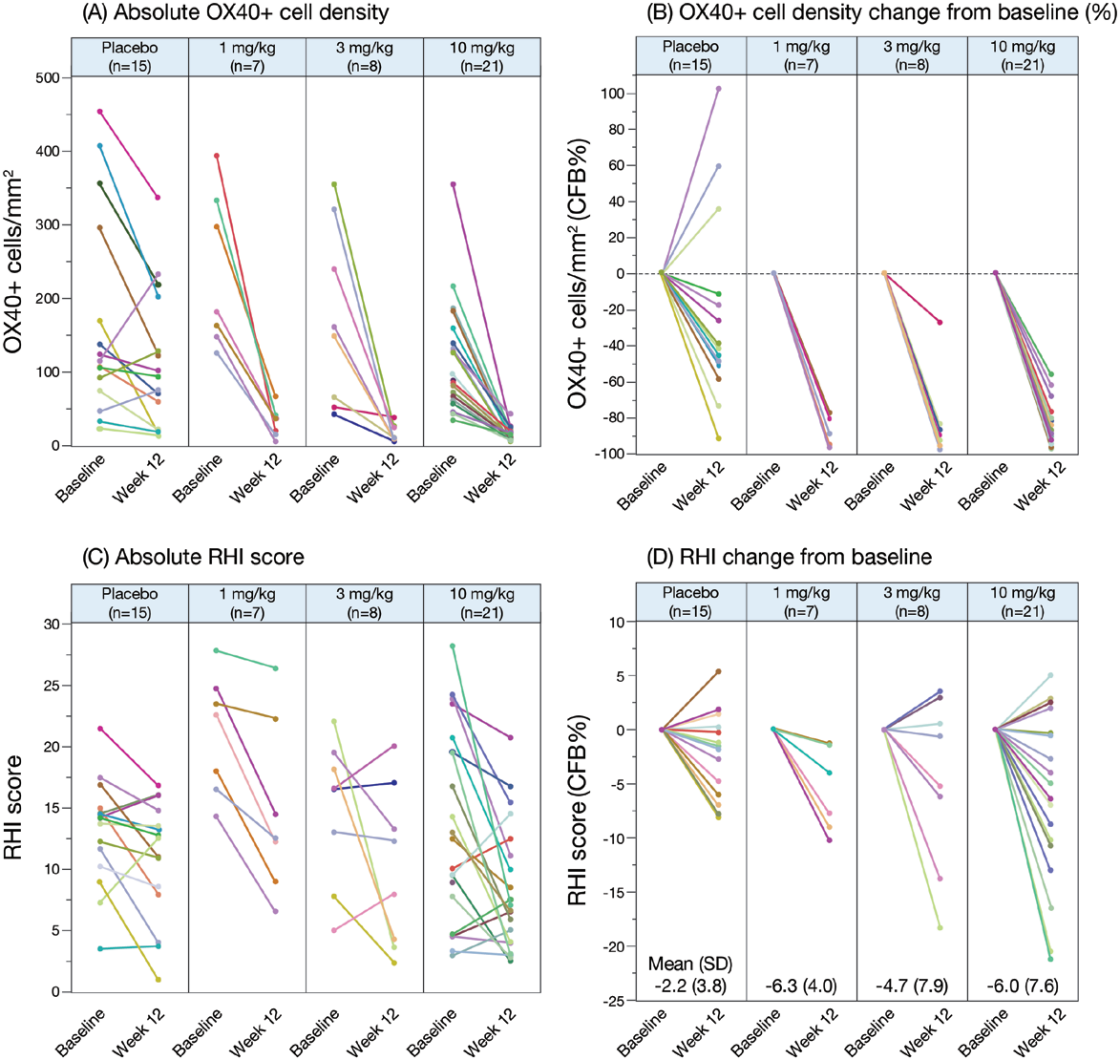
OX40+ cell density and RHI score by treatment at baseline and week 12 for individuals completing 12 weeks’ treatment: (A) absolute OX40+ cell density, (B) change in OX40+ cell density from baseline, (C) absolute RHI score, and (D) change in RHI score from baseline. Each point represents the mean of multiple (up to 4) tissue biopsy specimens for each patient. CFB, change from baseline.

**Figure 4.**
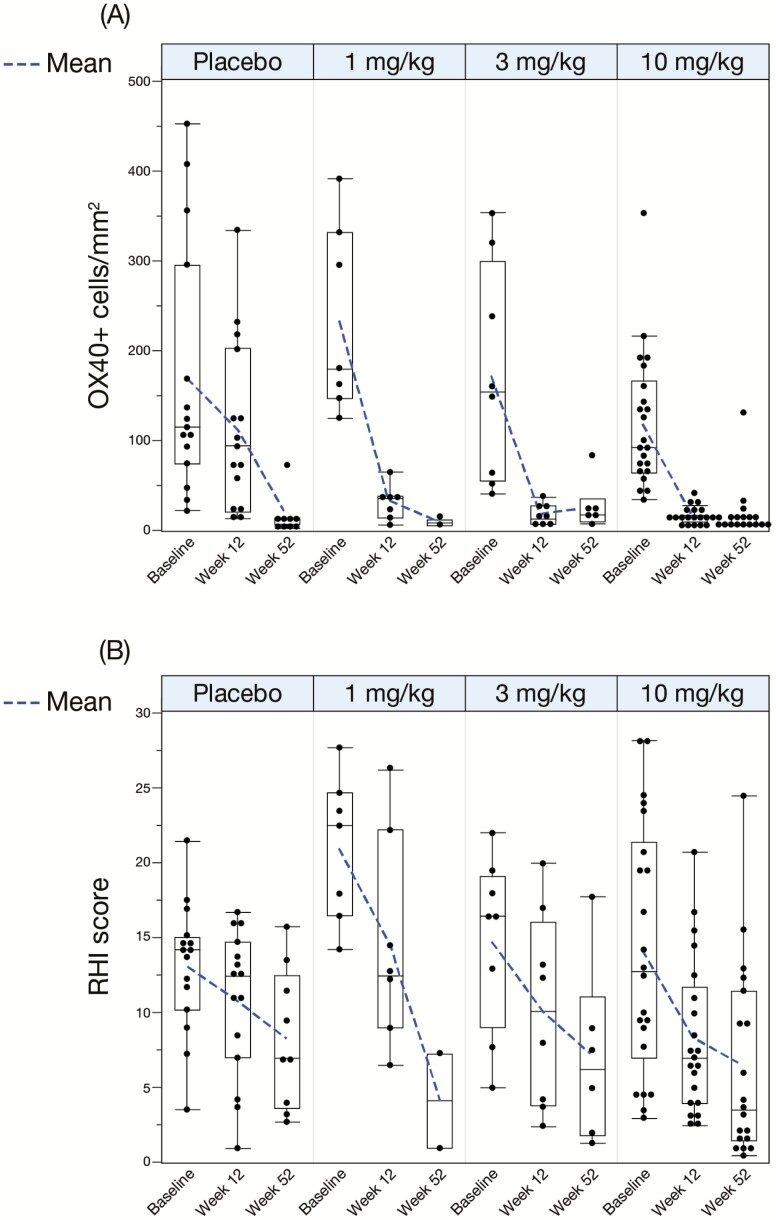
Box plots of (A) OX40+ cell density and (B) RHI score by treatment at baseline, week 12 and week 52 for individuals completing 12 weeks’ treatment. Each point represents the mean of multiple (up to 4) tissue biopsy specimens for each patient. *P* values (nominal) comparing KHK4083 versus placebo at week 12 (Tukey’s test).

**Figure 5.**
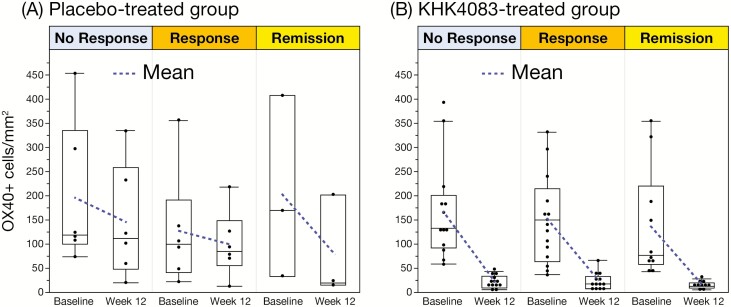
Box plots of OX40+ cell density at baseline and week 12 by clinical response in (A) placebo and (B) combined KHK4083 treatment groups for patients completing week 12.

All patients treated with KHK4083 regardless of dose showed a decrease in the OX40+ cell density at week 12 compared with baseline, whereas 3 of 15 placebo-treated patients showed an increase (Fig. 3A, B). RHI score improved more with each KHK4083 dose compared with placebo by week 12 (Fig. 3C, D), although there was no statistically significant difference in this relatively small population (Fig. 3C, D). The box plot of OX40+ cell density showed clear reduction by week 12 with almost complete elimination by week 52 for each KHK4083 dose, while the reduction with placebo was modest: furthermore, OX40+ cell density reduction at week 12 was statistically significant for each KHK4083 dose compared with placebo (Fig. 4A). OX40 levels were also significantly decreased at week 52 compared with week 12 in the patients who initially received placebo and were switched to KHK4083 at week 12 (Fig. 4A). The box plot of RHI score showed a decreasing trend over time regardless of treatment (KHK4083 or placebo) with no dose dependency over time (Fig. 4B). There was no clear correlation between the decrease in OX40+ cell density and clinical response at week 12 in either the placebo- or KHK4083-treated (all doses combined) group (Fig. 5A, B).

Among the 31 cytokine and chemokines tested, 3 (IL6, interferon-γ, and IL7) showed a statistically significant difference between nonresponders and those who showed a response/remission in KHK4083-treated patients for at least 1 time point at week 12 or 52 (data not shown). Serum IL6 and interferon-γ were decreased in both KHK4083- and placebo-treated patients who had response/remission compared with nonresponders. Serum IL7 decreased or remained stable in KHK4083-treated patients but increased in placebo-treated patients in those who had a response/remission. These results should be interpreted cautiously given the large number of comparisons that were performed. No clear dose dependency was observed for any cytokine/chemokine (data not shown).

## DISCUSSION

We examined the effects of 3 doses of IV KHK4083 (1, 3, and 10 mg/kg) every 2 weeks in a phase 2, randomized, placebo-controlled, parallel-group, double-blind, 12-week trial in patients with moderately active UC. KHK4083 was well tolerated with mild or moderate infusion-related reactions being the most frequent treatment-related AE, all of which occurred early during treatment (from baseline to week 12) and not during long-term extension (weeks 12–52). The number of patients who developed anti-KHK4083 antibodies was higher than expected given that KHK4083 is a fully human monoclonal antibody: furthermore, the KHK-4083 blood level was not affected in the single patient who developed neutralizing antibodies.

Exploratory analysis confirmed OX40+ cell depletion in the colonic mucosa of the patients who received KHK4083. Previous studies have consistently shown overexpression of OX40 in UC patients with active disease.^11^ Based on this finding, target engagement as well as proof of pharmacodynamic action for KHK4083 was confirmed in this trial.

In the serum cytokine and chemokine analysis, significant reductions in serum IL-6 and interferon-γ concentrations were observed in response/remission groups compared with nonresponders in both KHK4083- and placebo-treated patients, which suggests that T-helper type 1 cytokines are valid biomarkers of the inflammatory process. Further studies using a larger sample size would be required to make a definitive conclusion. On the other hand, there was no clear correlation between OX40+ cell depletion and clinical response, suggesting that the presence of OX40+ cells might not be a crucial factor for patients with established disease. In fact, switching from placebo to KHK4083 at week 12 did not make a significant difference in long-term efficacy at week 52, although the target cells were completely depleted. However, this finding must be reconciled with our observation that OX40+ cell density correlates with disease severity scored by endoscopy, which may suggest that OX40+ cells are relevant in the pathogenesis of UC. The contradiction in logic presented by our finding that depletion of OX40+ cells by KHK4083 was ineffective suggests that, although the pathological immune response is initiated through T-cell activation that may involve the OX40 pathway, as the disease progresses, factors other than the OX40+ T cell may also play a role in the perpetuation of inflammation.

There was no statistically significant difference between any KHK4083 dose group and placebo for LS mean change from baseline to week 12 in mMES, the prespecified primary end point for efficacy. Similarly, there were no significant differences between any of KHK4083 groups and placebo in the secondary efficacy end points (including total Mayo score, UCEIS, and clinical response, clinical remission, and mucosal healing rates). We additionally performed a post hoc retrospective analysis to explore the increase of statistical power of the observations using a newly developed composite scoring system, UC-100, which is comprised of clinical, endoscopic, and histological measures.^18^ However, UC-100 data did not show any significant efficacy with any dose of KHK4083 compared with placebo treatment (data not shown).

## CONCLUSIONS

The IV administration of KHK4083 at doses up to 10 mg/kg every 2 or 4 weeks appeared safe during long-term administration up to 52 weeks. KHK4083 depleted OX40+ cells in the colonic mucosa, constituting proof of pharmacodynamic action. KHK4083 did not show improvement in clinical response or mucosal healing compared with placebo, but the sample size was small and the placebo response was relatively high. Conflicting results were also seen for clinical response and mucosal healing when analyzed by RHI stratification of less and more severe patients at baseline and, furthermore, indicated a potential imbalance in baseline RHI. Based on these data, a larger study and/or a study in a population with high RHI may be required to quantify definitively the relationship between clinical response to KHK4083 and depletion of OX40+ cells in patients with UC.

## Supplementary Material

otaa049_suppl_Supplementary_Material

## Data Availability

The authors confirm that the data supporting the findings of this study are available within the article and/or its Supplementary Material.

## Notes

Author Contribution: V.S. takes responsibility for the integrity of the work as a whole, from inception to published article. Patient recruitment and acquisition of data (Ja.K., M.P., M.K., and M.B.), protocol design (N.K., S.E., Je.K., Y.N., C.J., T.M., B.G.F., and V.S.), study management (C.J., T.M., and V.S.), data analysis (N.K., C.J., T.M., Y.N., B.G.F., and V.S.), data collection (N.K. and V.S.), medical monitor (S.E.), pharmacokinetic analysis (Je.K.), data management (Y.N.), and scientific advisor (B.G.F.). All authors provided critical review of the manuscript and approved the final version of the manuscript.

Funding: This work was supported by Kyowa Kirin Pharmaceutical Development, Inc., Princeton, NJ.

Conflict of Interest: S.E., Je.K., Y.N., C.J., N.K., T.M., and V.S. are employees of Kyowa Kirin Pharmaceutical Development, Inc., Princeton, NJ. B.G.F. is a consultant to Ablynx, Progenity, NextBiotix, Gossamer Pharma, Qu Biologics, Sienna Biologics, Parimmune, BioMx Israel, Boston Pharmaceuticals, AdMIRx Inc, Akebia Therapeutics Inc., Mesoblast Pharma, Millennium, Nestles, Novo Nordisk, Novartis, Pfizer, Promethus Therapeutics, Protagonist, Receptos, Salix, Shire, Sigmoid Pharma, Synergy Pharma, Takeda, Teva Pharma, TiGenix, Tillots, UCB, Vertex, VHsquared, Wyeth, Zealand, Galapagos, Inception, IBD Inc., Lexicon, Vivelix Pharma and Japan Tobacco Company, and Senior Scientific Officer at Robarts Clinical Trials. Ja.K., M.P., M.K., and M.B. have no conflicts of interest to declare.
