# Corrigendum to: Randomized, Ascending Dose, Phase 2 Study of KHK4083, an Anti-OX40 Monoclonal Antibody, in Moderately Active Ulcerative Colitis

**DOI:** 10.1093/crocol/otaa092

**Published:** 2020-12-17

**Authors:** Jaroslaw Kierkus, Marina Pesegova, Maria Klopocka, Marija Brankovic, Noriyuki Kasai, Sergey Efuni, Jennifer Kong, Yu Nakajima, Christina Jordan, Takeshi Matsui, Brian G Feagan, Vincent Strout

In the originally published version of this manuscript, several errors were noted and listed in this corrigendum.

Upon the original publication, author Maria Klopocka, MD’s affiliation was incorrect. The affiliation should read: “Department of Gastroenterology and Nutrition, Nicolaus Copernicus University in Toruń, Collegium Medicum in Bydgoszcz, Poland”.

Upon the original publication, Table 1 indicated inaccurate “Placebo” results in the following cells: “Colitis ulcerative”, “Pyrexia”, “Arthralgia”. Table 1 also indicated inaccurate “All patients” results in the following cells: “Any treatment-emergent AE”, “Colitis ulcerative”, “Pyrexia”, “Arthralgia”.

These have now been corrected online.

