# Supplementary material for: Randomized, Ascending Dose, Phase 2 Study of KHK4083, an Anti-OX40 Monoclonal Antibody, in Moderately Active Ulcerative Colitis
Source: Crohns Colitis 360. 2020 Jun 9;2(3):otaa049. doi: 10.1093/crocol/otaa049 (PMC9808818; doi:10.1093/crocol/otaa049)

SUPPLEMENTARY INFORMATION

Randomized, Ascending Dose, Phase 2 Study of KHK4083, an Anti-OX40 Monoclonal Antibody, in Moderately Active Ulcerative Colitis

Jaroslaw Kierkus, Marina Pesegova, Maria Klopocka, Marija Brankovic, Noriyuki Kasai, Sergey Efuni, Jennifer Kong, Yu Nakajima, Christina Jordan, Takeshi Matsui, Brian G. Feagan and Vincent Strout

CONTENTS

List of Investigators..3
Methods...4
Protocol Amendments...4
Inclusion Criteria...5
Exclusion Criteria..6
Rationale for Dose Selection...9
Objectives...10
Results...12
Tables..13
Table 1. Demographic and Baseline Clinical Characteristics…………………………13
Table 2. UCEIS Change at Week 12..14
Table 3. Total Mayo Change at Week 12...15
Table 4. Clinical Response, Clinical Remission, and Mucosal Healing Rates at Week 12……………………………………………………………………………………...16
Table 5. Endoscopic Healing Rates according to Total Mayo Score.. 17
Table 6. KHK4083 Pharmacokinetic Parameters...18
Figures...19
Figure 1. Effect of KHK4083 on T-cell Activation and Memory Cell Expansion via OX40 Blockade……………………………………………………………………...19
Figure 2. OX40-Stained Cells...20
Figure 3. Correlation between Baseline RHI Score and Clinical Response at Week 12. ……………………..………………………………………………………..21
Figure 4. CONSORT Diagram...22
Figure 5. Serum KHK4083 Concentrations...23

LIST OF INVESTIGATORS

Principal investigator	Affiliation
Suzy Kim, MD	Gastroenterology Associates, PA, Greenville, SC, USA
Robert Sike, MD	Department Gastroenterology, Szent Margit Hospital, Budapest, Hungary
Peter Lakatos, MD	Department of Internal Medicine I, Semmelweis University, Budapest, Hungary
Gabor Tamas Toth, MD	1st Department of Internal Medicine, Gastroenterology, Szent Janos Hospital and Unified Hospitals of North Buda, Budapest, Hungary
Jaroslaw Kierkuś, MD, PhD	Gastroenterology Diagnostic Facility for Adults, Maternal, Pediatric and Adolescent Healthcare Centre, Warsaw, Poland
Prof. Maria Klopocka, MD, PhD	Jan Biziel University Hospital #2, Bydgoszczy, Poland
Robert Petryka, MD	Niepubliczny Zaklad Opieki Zdrowotnej VIVAMED Jadwiga Miecz, Warsaw, Poland
Marek Horyński, MD, PhD	Endoskopia Spólka z ograniczoną odpowiedzialnością, Sopot, Poland
Tomasz Romańczyk, MD, PhD	H-T. Centrum Medyczne Sp. z o,o, Spólka Komandytowa, Thchy, Poland
Jerzy Rozciecha, MD, PhD	Lexmedica Clinical Research Centre, Wroclaw, Poland
Bartosz Korczowski, MD, PhD	Korczowski Bartosz, Gabinet Lekarski, Rzeszów, Poland
Assoc. Prof. Radu Bogdan Mateescu, MD	Department of Gastroenterology, Colentina Clinical Hospital, Bucharest, Romania
Irina Kasherininova, MD	City General Hospital #2, St. Petersburg, Russia
Prof. Elena A. Belousova, MD	Gastroenterology Department, Moscow Vladimirsky Regional Clinical Research Institute, Moscow, Russia
Prof. Konstantin P. Zhidkov, MD	City Hospital #26, St. Petersburg, Russia
Prof. Marina F. Osipenko, MD	City Clinical Hospital #13, Novosibirsk, Russia
Prof. Marina Pesegova, MD	Department of Gastroenterology, Territorial Clinical Hospital, Krasnoyarsk, Russia
Natalia Galkina	State Budget Medical Institution, N.N. Burdenko Penza Regional Clinical Hospital, Penza, Russia
Prof. Predrag Dugalic, MD, MSc	Department of Gastroenterology, Clinical Hospital Center Zemun, Belgrade, Serbia
Asst. Prof. Natasa Zdravkovic Pertovic, MD, PhD	Kragujevac Clinical Center, Kragujevac, Serbia
Dr. Marija Brankovic, MD, MSc	Department of Gastroenterology, Clinical Hospital Centre Bezanijska Kosa, Belgrade, Serbia
Vladimir Knoflicek, MD	Department of Internal Medicine, Znojmo Hospital, Znojmo, Czech Republic
Miroslava Volfova, MD	Hepato-Gastroenterology HK Ltd., Hradec Kralove, Czech Republic

METHODS

Protocol Amendments

Two sets of amendments were made to the protocol. The following key changes were made in Amendment 1 (15 Oct 2015):

- The primary objective/variable was changed from the Ulcerative Colitis Endoscopic Index of Severity (UCEIS) score to the Food and Drug Administration (FDA) suggested modified Mayo endoscopy subscore (mMES) with an amended endoscopy finding scoring.
- The secondary objective/variable was changed from Mayo endoscopy subscore to UCEIS.
- The exploratory objective/variable of a more stringent definition of remission defined as an mMES of 0 or 1, a stool frequency subscore of 0 or 1, and a rectal bleeding subscore of 0 was added.
- The minimal dose of budesonide was increased from 6 to 9 mg/day in the definition of an unsuccessful prior treatment.
- The minimal doses and duration of tumor necrosis factor alpha (TNFα] antagonists in the definition of an unsuccessful prior treatment were specified.
- Clarification of when the investigator(s) may withdraw a patient from the study.
- Defined unacceptable overall safety risks that may result in recommendation for termination of the study.
- Clarification of additional reasons that a patient may be removed from the study.
- Clarified that daily, chronic antidiarrheal medications should not be taken.
- The handling of investigational product administration when visits were not within the study visit window was clarified.
- A separate subsection of definitions of infusion reaction intensity (ie, mild, moderate, and severe) and a separate subsection to describe the reporting of infusion reaction adverse events (AEs) was provided.
- Mayo Scoring System for assessment of ulcerative colitis activity (score 0–12) with mMES was amended to exclude the appearance of mild friability resulting in a score of 1.
- A reference for the clinical diagnostic criteria of anaphylaxis as per the US FDA Guidance for Industry Immunogenicity Assessment for Therapeutic Protein Products 2014 was created.

The following key changes were made in Amendment 2 (4 Oct 2016):

- The study plan and objectives were revised to include an open-label extension. The post-induction phase of the study was changed from a blinded, long-term extension to an open-label extension and eligibility was not based on clinical response or mucosal healing.
- All eligible patients were allowed to receive KHK4083 after the 12-week, double-blind induction phase. Patients in the long-term extension who were receiving study medication but were non-responders up to the week 28 visit were eligible to receive KHK4083 in the open-label study extension.
- Investigative sites in the Czech Republic were included.

- The safety monitoring plan was changed to evaluate the cumulative safety data and dose reductions or no dose escalations were allowed.
- Patients with well-controlled immunological, autoimmune or chronic inflammatory disorders, other than ulcerative colitis (UC), or autoimmune connective tissue diseases were allowed in the study.
- Patients receiving stable doses of oral budesonide ≤9 mg/day were allowed to participate.
- The criteria for patient removal from treatment and the study was updated to include patients who had worsening in Mayo Clinic score during induction therapy.
- Clarification of sigmoidoscopy times for patients who discontinued during induction therapy was provided.
- The pharmacokinetic and pharmacodynamic sampling schedule was modified.

Inclusion Criteria

Patients were included if all of the following inclusion criteria were fulfilled:

1) Patient was able and willing to comply with study procedures, and to adhere to dosing and visit schedules and follow-up procedures as described in the protocol and informed consent form;
2) Patient voluntarily signed/dated an Institutional Review Board/Independent Ethics Committee-approved informed consent form in accordance with regulatory and institutional guidelines;
   (Note: Written informed consent was obtained prior to performing any study-related procedure.)
3) Male and female patients ≥18 years of age at the time of enrolment;
4) Patient had UC that was diagnosed >6 months prior to the screening visit;
5) Patient had moderately active UC defined as:
   a) Total Mayo score of 4–9 (range 0–12, with higher scores indicating more disease activity);
   b) Endoscopy subscore (mMES determined by a central reader) >2; and
   c) Disease that extended ≥15 cm from the anal verge.
6) Patient had previous treatment (<5 years prior to screening) with one or more of the following: corticosteroids, immunosuppressive medications, or TNFα antagonist therapy that was unsuccessful because of a lack of efficacy response or AEs, as defined below:
   a) corticosteroids for induction therapy of at least prednisolone-equivalent of 20 mg (or oral budesonide 9 mg) oral daily for 2 weeks or injectable for 1 week, or for maintenance therapy at least two failed attempts to reduce to less than prednisolone-equivalent 10 mg (or oral budesonide 3 mg) oral daily, or a history of intolerance to corticosteroids (including but not limited to hypertension, insomnia, osteopenia, osteoporosis, hyperglycemia, infection, or Cushing's syndrome);
   b) azathioprine or 6-mercaptopurine ≥1.5 mg/kg/day or ≥0.75 mg/kg/day, respectively, for 8 weeks, or a history of intolerance to either agent (including but not limited to nausea, vomiting, abdominal pain, aspartate aminotransferase (AST) or alanine aminotransferase (ALT) elevations, thiopurine methyltransferase genetic mutation, or infection);

c) TNFα antagonists for induction therapy with approved anti-TNFα products including, but not limited to, infliximab 5 mg/kg intravenously (IV) for 2 doses ≥2 weeks apart, adalimumab 160 mg subcutaneously (SC) followed by ≥80 mg SC ≥2 weeks apart, and golimumab 200 mg SC followed by ≥100 mg ≥2 weeks apart and anti-TNFα biosimilar products with approved dosages for 2 doses ≥2 weeks apart; or as maintenance therapy for recurrence of symptoms despite continued dosing or history of intolerance (including but not limited to infusion or injection reactions, demyelination or infection).

7) Female patients who were considered to be women of child-bearing potential had a negative pregnancy test at screening and baseline. Women of child-bearing potential agreed to use effective contraception defined as oral contraceptives with one barrier method or tubal ligation with one barrier method or double barrier method (condom plus spermicide or diaphragm plus spermicide) during the study and for ≥6 months after the last dose of investigational product. Patients were considered to not be of child-bearing potential if they were ≥50 years of age and without menses for 24 consecutive months and had a follicle-stimulating hormone level >25 mIU/mL (or in postmenopausal range per local laboratory standards) or had undergone a hysterectomy and/or a bilateral salpingo-oophorectomy. Egg donation was not permitted while on study medication and for ≥6 months after the last dose of study medication.
8) Male patients (including those who have had a vasectomy) used adequate contraception (eg, latex condom, non-latex condom not made of natural animal membrane such as polyurethane condom) during the study and for ≥6 months after the last dose of investigational product. Sperm donation was not permitted while on study medication and for ≥6 months after the last dose of study medication.

Exclusion Criteria

Patients were excluded from the study for any of the following reasons:

1) Patient, who, for any reason, was judged by the investigator to be inappropriate for this study, including a patient who was unable to communicate or cooperate with the Investigator, who had a psychiatric illness, disability or social situation that may compromise the safety of the patient during the study or affect the ability of the patient to adhere to study procedures;
2) Patient had a medical history of clinically significant (as determined by the investigator or the sponsor) cardiac, renal, hepatic/biliary (eg sclerosing cholangitis), pulmonary or other medical conditions, or was not generally in good health.
3) Patients with the history of immunological, autoimmune, or chronic inflammatory disorders (eg, uveitis, rheumatoid arthritis, ankylosing spondylitis or spondyloarthritis, psoriasis) other than UC or autoimmune connective tissue diseases (eg, systemic lupus erythematosus, systemic sclerosis) and were well controlled may have been included into the trial after consultancy with medical monitor. Patients with thyroid disorders, vitiligo and alopecia were eligible for inclusion.

4) Patient's UC had failed to respond to:
   a) Two or more biologic treatments with different mechanisms of action (eg, infliximab, vedolizumab) or
   b) Three or more anti-TNFα biologics, eg, infliximab, adalimumab, golimumab;
5) Patient required prescription treatment for UC, except for the stable, oral treatment of UC as follows:
   a) Aminosalicylates (5-aminosalicylic acid [5-ASA] or mesalamine ≤4.8 g/day; sulfasalazine ≤3 g/day) for ≥ 14 days prior to the screening visit; and/or
   b) Glucocorticoids (≤20 mg/day prednisolone or the equivalent, or budesonide ≤9 mg/day) for ≥14 days prior to the screening visit (or for 4 weeks if a course of corticosteroids had started at <8 weeks prior to screening visit) and/or
   c) Azathioprine ≤3 mg/kg/day or 6-mercaptopurine ≤1.5 mg/kg/day for a total treatment period of ≥12 weeks, including 4 weeks of stable treatment, prior to the screening visit.
6) Patient had received any of the following prior treatments or treatments within the specified time:
   a) Natalizumab, efalizumab, rituximab, or other lymphocyte-depleting treatments including but not limited to alkylating agents (such as cyclophosphamide or chlorambucil) and total lymphoid irradiation at any time prior to randomization (baseline visit);
   b) TNFα antagonists within 8 weeks, or 5 half-lives (based on maximum duration but not exceeding 12 weeks) prior to randomization (baseline visit);
   c) Vedolizumab within 16 weeks prior to randomization (baseline visit);
   d) Methotrexate, cyclosporine, mycophenolate, tacrolimus, thalidomide, or other immune-altering drugs within 4 weeks prior to randomization (baseline visit) (ophthalmological preparations were permitted);
   e) 5-ASA enema, or steroid enema or suppository use within 2 weeks prior to randomization (baseline visit) and/or
   f) Investigational agents within 8 weeks or 5 half-lives (if pharmacology information is available) prior to randomization (baseline visit) whichever was longer.
7) Patient with recent (within 1 year prior to screening), suspected or confirmed symptomatic stenosis of the colon, abdominal abscess or ischemic colitis based on clinical or radiographic data; or who had suspected, confirmed or a history of toxic megacolon; or with any colonic resection, subtotal or total colectomy, ileostomy or colostomy; or who had any previous surgery for UC or an anticipated requirement for surgery for UC;
8) Patient with known colonic dysplasia, adenomas, or polyposis;
9) Patient had major surgery within 4 weeks prior to screening or an anticipated requirement for major surgery;
10) Patient with enteric pathogens (including *Clostridium difficile*) detected on stool analysis; or *C. difficile* infection within 8 weeks prior to screening; or intestinal pathogen infection detected within 4 weeks prior to screening;
11) Patient with any of the following hematological and chemistry laboratory values:
   a) Platelet count <100,000/mm^3;
   b) Neutrophils <1500/mm^3;

c) Serum creatinine ≥1.6 mg/dL (≥144.4 μmol/L);
d) Alkaline phosphatase >3 times the upper limit of normal (ULN);
e) AST or ALT >2 times ULN;
f) Total bilirubin >2 mg/dL, unless due to Gilbert's syndrome;
g) Serum albumin <3 g/dL;
h) Hemoglobin <9 g/dL;
i) Glycated serum hemoglobin $A1_c$ ≥9%.

12) Patient had clinically significant cardiac disease (class II, III, or IV of the New York Heart Association classification); unstable angina pectoris; myocardial infarction within 6 months or was post-angioplasty or stenting within 6 months; uncontrolled hypertension; or clinically significant abnormality, such as cardiac arrhythmia, on a 12-lead electrocardiogram at screening;
13) Patient was pregnant or breast-feeding;
14) Patient had major immunological reaction (eg, serum sickness, anaphylaxis, anaphylactoid reaction);
15) Patient was hepatitis B core antibody or surface antigen positive at screening and/or hepatitis C antibody positive with detectable RNA at screening;
16) Patient had a history of HIV positivity, tests positive for HIV at screening, or had congenital or acquired immunodeficiency;
17) Patient had active tuberculosis (TB), suspected extra-pulmonary TB, a history of incompletely treated TB, or latent TB or other latent infection. Patients with latent TB (purified protein derivative [PPD] or interferon gamma-release assay [IGRA]) could be included in the study if prophylactic therapy for latent TB was started >4 weeks prior to screening. Patients with a potentially untreated other infection (clinical findings) were excluded;
(Note: Performing of both IGRA and PPD tests in the same patient was avoided as screening procedures. Positivity of one of the tests cannot be voided by negativity of the other, ie, patients with positive PPD and negative IGRA test performed as a part of screening were considered TB positive and were required to start prophylactic anti-TB therapy prior to infusion of investigational product according to local treatment standards. All safety laboratory tests could be repeated. Screening procedures could be extended by 2 weeks.)
18) Patient had bacterial infections requiring treatment with oral or parenteral antibiotics (topical antibiotics are allowed) within 2 and 4 weeks, respectively, of the screening period;
19) Patient had a history of systemic opportunistic infection or recurrent infections;
20) Patient had malignancy or history of malignancy except for adequately treated basal cell skin cancer or adequately treated carcinoma in situ of the cervix without recurrence, and treatment was completed ≥5 years before the screening period;
21) Patient who received a bacillus Calmette-Guérin vaccine within 6 months of randomization or live vaccination (eg, measles/mumps/rubella, herpes zoster, varicella, intranasal influenza, oral poliomyelitis) within 4 weeks of randomization was excluded. Patient was allowed vaccinations of inactivated vaccines (eg, hepatitis, pneumococcal, meningococcal, tetanus, diphtheria toxoid, acellular pertussis, inactivated polio, human papilloma, influenza apart from intranasal influenza);

22) Patient with a history of substance abuse within 1 year of screening; or active marijuana (medicinal or recreational) use or active substance abuse;
23) Patient had other severe acute or chronic medical or psychiatric condition or laboratory abnormality that increased the risk associated with study participation or investigational product administration, or interfered with the interpretation of study results as determined by the investigator;
24) Patient who previously participated in a study of KHK4083.
(Note: A patient who was considered a screen failure and not dosed with investigational product in the current study was permitted to be re-screened once due to failure to meet the inclusion/exclusion criteria, whereas patients with other reasons for lack of randomization could be re-screened twice.)

Rationale for Dose Selection

In the first human phase 1, single-dose study of KHK4083 in 68 patients with plaque psoriasis (Papp et al. 2017), dose escalation of KHK4083 was started at 0.003 mg/kg (cohort 1) followed by single ascending dose cohorts of 0.01, 0.03, 0.1, 0.3, 1.0, and 3.0 mg/kg (cohorts 2–7) administered as 60-min IV infusions. A single subcutaneous dose of 1.0 mg/kg (cohort 8) was also administered. This study was amended to include an additional single ascending dose cohort of 10 mg/kg (cohort 9) administered as a 120-min IV infusion. The doses selected for the current study were based on the phase 1 study, which showed that KHK4083 was well tolerated and there were no dose-limiting adverse effects at doses ≤10 mg/kg.

The pharmacokinetic data from IV dose cohorts 1–7 and cohort 9 were analyzed and trial simulations were performed for the present phase 2, multiple ascending dose study of KHK4083 for administration every 2 or 4 weeks at 1.0, 3.0, and 10 mg/kg as a 60-min (± 10 min) IV infusion. In a 26-week Good Laboratory Practice (GLP) toxicology study in male and female cynomolgus monkeys at the no-observed -adverse-effect level (NOAEL) of 30 mg/kg (unpublished data on file, Kyowa Kirin Pharmaceutical Development, Inc.), the mean area under the serum concentration-time curve from time 0 to week 2 ($AUC_{0–2wk}$), and maximum serum concentration (C_{max}) were 301,500 µg•hr/mL and 1,510 µg/mL, respectively, after the 13th dose (ie, steady state). The safety margins ($AUC_{0–2wk}$ from the monkeys and NOAEL/$AUC_{0–2wk}$ simulated in humans) were 48.4, 10.3, and 1.9 at 1.0, 3.0, and 10 mg/kg, respectively. In a 4-week GLP toxicology study in male and female cynomolgus monkeys at the NOAEL of 100 mg/kg (unpublished data on file, Kyowa Kirin Pharmaceutical Development, Inc.), the mean area under the serum concentration-time curve from time 0 to infinity ($AUC_{0–\infty}$) and C_{max} were 607,000 µg·hr/mL and 5,345 µg/mL, respectively, after the first dose. The safety margins ($AUC_{0–\infty}$ from the monkey NOAEL/$AUC_{0–2wk}$ simulated in humans) were 97.4, 20.8 and 3.8 at 1.0, 3.0 and 10 mg/kg, respectively. The safety margins calculated based on C_{max} were comparable to the safety margins calculated based on $AUC_{0–2wk}$. As the phase 2 study was to reach the pharmacokinetic exposure at the NOAEL dose level, and the 1.0, 3.0, and 10 mg/kg doses given every 2 or 4 weeks were expected to have overall pharmacokinetic exposure lower than the NOAEL, the selected doses were expected to be a safe for this clinical study.

Objectives

The primary, secondary (including pharmacokinetics and immunogenicity) and exploratory objectives of the study were as follows:

Primary

- Induction therapy – part A: To determine the safety and tolerability of administration of multiple ascending doses of KHK4083 and to select the highest dose tolerated by patients with moderately active UC to recommend for use in part B;
- Induction therapy – part B: To determine if the recommended dose of KHK4083 identified in part A improves the mucosa in patients with moderately active UC at week 12 as measured by the mMES.

The assessment was based on a mean change in the mMES (subscores from 0 to 3 with modified endoscopy finding scoring, ie, excluding mild friability from a subscore of 1 from baseline (week 0) to week 12.

Secondary

To determine if KHK4083 at dose levels different than the recommended dose improve the mucosa based on the mMES;

- To determine if any dose level of KHK4083 administered as induction therapy met the following objectives at week 12 (or as noted):
  - Improve the mucosa based on the modified Baron endoscopic score;
  - Improve the mucosa based on the UCEIS;
    The assessment was based on a mean change in the UCEIS (scores from 0 to 8 based on findings of vascular pattern, bleeding, and erosions/ulcers) from baseline (week 0) to week 12;
  - Induce mucosal healing based on the mMES;
  - Improve clinical signs and symptoms based on total Mayo score;
  - Improve clinical signs and symptoms based on partial Mayo scores (week 2 to week 12, excludes endoscopy subscores);
  - Induce a clinical response based on a reduction in the total Mayo score (ie, reduction of ≥3 points and a decrease of ≥30% from baseline (week 0) to week 12) and rectal bleeding subscale (ie, reduction of ≥1 point from baseline (week 0) to week 12) (or a defined absolute rectal bleeding score of 0 or 1 at week 12);
  - Induce clinical remission based on a total Mayo score (ie, score of ≤2) and subscores (ie, no subscores >1).
- To characterize the pharmacokinetics of KHK4083 in patients with moderately active UC following multiple dose administration;
- To evaluate the development of antibodies against KHK4083 (immunogenicity).

Exploratory

- To determine if multiple doses of KHK4083 administered during the open-label extension (OLE)/long-term extension (LTE) therapy phase met the following objectives at week 52 (or as noted) when compared with baseline (week 0) scores (or subscores) or assessments:

- Improve clinical signs and symptoms based on total Mayo score;
  - Improve clinical signs and symptoms based on partial Mayo scores (excludes endoscopy subscores) at week 16 to week 52 and the LTE therapy follow-up period (week 56 to week 64);
  - Induce a clinical response based on a reduction in the total Mayo score and rectal bleeding subscale (or a defined absolute rectal bleeding score);
  - Induce clinical remission based on a total Mayo score and subscores;
  - Induce durable clinical responses and durable clinical remissions (present at both weeks 12 and 52) and glucocorticoid-free clinical remission;
  - Induce mucosal healing based on the mMES;
  - Improve the mucosa based on the mMES, UCEIS, and/or modified Baron endoscopic score;
  - Induce remission (week 12; week 52) based on modified Mayo endoscopy, stool frequency, and rectal bleeding subscores (defined as an mMES of 0 or 1, stool frequency subscore of 0 or 1, and rectal bleeding subscore of 0);
- To evaluate the activity of KHK4083 on health-related quality of life, which was based on the patients' completed Inflammatory Bowel Disease Questionnaires compared with baseline assessments;
- To measure changes from baseline in corticosteroid (glucocorticoid) dosages;
- To determine the percentage of patients who were glucocorticoid-free from week 16 to week 52 and through the OLE/LTE therapy follow-up period (week 56 to week 64);
- To evaluate the pharmacodynamic profile of KHK4083;
- To explore the pharmacokinetic-pharmacodynamic relationships.

Reference

Papp KA, Gooderham MJ, Girard G, et al. Phase I randomized study of KHK4083, an anti-OX40 monoclonal antibody, in patients with mild to moderate plaque psoriasis. *J Eur Acad Dermatol Venereol.* 2017;31:1324–1332.

Results

Pharmacokinetic data are presented in Supplementary Table 5 and Supplementary Figure 3. Mean serum concentrations of KHK4083 increased dose dependently. Mean terminal elimination half-life (8.7–17.6 days) showed no obvious relationship to dose or change over time. Exposure (as maximum serum concentration and area under the serum concentration-time curve [AUC]) to KHK4083 was proportional to dose over the range from 1 to 10 mg/kg. The median accumulation ratio of the AUC from time zero over the dose interval ($AUC_{0-\tau}$) at week 10 was 2.2, 2.17, and 1.69 at 1, 3, and 10 mg/kg of KHK4083, respectively, as expected from the half-life of KHK4083.

Anti-KHK4083 antibodies were positive at baseline in one (11.1%), two (20.0%), and one (3.3%) patients receiving KHK4083 1, 3, and 10 mg/kg, respectively, and were positive after treatment in six (66.7%), two (20.0%), and five (18.5%) patients, respectively. One patient receiving KHK4083 1 mg/kg developed anti-KHK4083 neutralizing antibodies, which had no effect on their serum KHK4083 concentration-time profile.

TABLES

TABLE 1: Demographic and Baseline Clinical Characteristics (Safety Analysis Set)

	KHK4083					
	**Cohort 1 (1 mg/kg) (n = 9)**	**Cohort 2 (3 mg/kg) (n = 10)**	**Cohort 3 (10 mg/kg) (n = 9)**	**Cohort 4 (10 mg/kg) (n = 21)**	**Cohorts 1-4 (combined) (n = 49)**	**Placebo (n = 17)**
Age (years), mean (SD)	47.3 (17.3)	46.1 (17.8)	41.2 (16.1)	41.1 (16.8)	43.3 (16.6)	33.8 (13.0)
Male, n (%)	5 (55.6)	8 (80.0)	4 (44.4)	15 (71.4)	32 (65.3)	9 (52.9)
Race, n (%)						
White	9 (100.0)	10 (100.0)	9 (100.0)	21 (100.0)	49 (100.0)	16 (94.1)
Black	0	0	0	0	0	1 (5.9)
BMI (kg/m^2), mean (SD)	25.8 (3.1)	27.4 (3.7)	22.8 (4.1)	23.5 (4.3)	24.6 (4.2)	25.2 (5.1)
Time since UC diagnosis (years), mean (SD)	9.4 (11.0)	7.8 (7.0)	9.7 (6.3)	7.2 (5.3)	8.2 (7.0)	6.1 (3.4)
Total Mayo score, mean (SD)	7.4 (1.3)	7.4 (1.3)	7.3 (1.2)	7.8 (1.5)	7.6 (1.4)	7.4 (1.1)
Partial Mayo score, mean (SD)	4.4 (1.3)	4.6 (1.3)	4.4 (1.2)	5.1 (1.3)	4.8 (1.3)	4.8 (1.1)
mMES, n (%)						
2	0	2 (20.0)	1 (11.1)	7 (33.3)	10 (20.4)	7 (41.2)
3	9 (100.0)	8 (80.0)	8 (88.9)	14 (66.7)	39 (79.6)	10 (58.8)
Modified Baron endoscopic score, mean (SD)	4.0 (0.0)	3.7 (0.7)	3.7 (0.7)	3.3 (1.1)	3.6 (0.8)	3.2 (1.1)
UCEIS score, mean (SD)	5.1 (0.9)	5.0 (1.8)	5.3 (1.6)	4.9 (1.5)	5.0 (1.5)	4.6 (1.5)
Prior UC medication use, n (%)						
Systemic corticosteroid*	4 (44.4)	6 (60.0)	5 (55.6)	8 (38.1)	23 (46.9)	9 (52.9)
5-Aminosalicylic acid†	3 (33.3)	3 (30.0)	4 (44.4)	4 (19.0)	14 (28.6)	6 (35.3)
Local corticosteroid‡	0	2 (20.0)	0	4 (19.0)	6 (12.2)	4 (23.5)
TNFα antagonist§	0	1 (10.0)	4 (44.4)	2 (9.5)	7 (14.3)	5 (29.4)
Immunosuppressant¶	2 (22.2)	1 (10.0)	4 (44.4)	2 (9.5)	9 (18.4)	7 (41.2)

BMI indicates body mass index; mMES, modified Mayo endoscopy subscore; SD, standard deviation; TNF, tumor necrosis factor; UC, ulcerative colitis; UCEIS, Ulcerative Colitis Endoscopic Index of Severity.

*Includes prednisolone, methylprednisolone, prednisone, hydrocortisone and dexamethasone.

†Includes mesalazine and sulfasalazine.

‡Includes budesonide and hydrocortisone.

§Includes azathioprine and methotrexate.

¶Includes infliximab and adalimumab.

TABLE 2: UCEIS: Actual and Change from Baseline at Week 12 (Full Analysis Set)

	KHK4083 dose				
	**1 mg/kg (n = 7)**	**3 mg/kg (n = 8)**	**10 mg/kg (n = 22)**	**All doses (n = 37)**	**Placebo (n = 15)**
Baseline					
Mean (SD)	5.0 (0.6)	5.1 (2.0)	4.9 (1.7)	5.0 (1.6)	4.7 (1.6)
Week 12					
Mean (SD)	4.9 (2.0)	3.0 (2.2)	3.8 (2.7)	3.8 (2.5)	3.5 (2.3)
Change from baseline to week 12					
Mean (SD)	–0.1 (2.0)	–2.1 (2.0)	–1.1 (3.1)	–1.1 (2.7)	–1.1 (2.0)
95% CI	–2.0, –1.7	–3.8, –0.5	–2.4, 0.3	2.0, –0.2	–2.2, 0.0
LSM (SE)	–0.079 (0.894)	–1.992 (0.837)	–1.077 (0.504)	–	–1.254 (0.612)
95% CI of change	–1.877 to 1.719	–3.677 to –0.307	–2.091 to –0.063	–	–2.485 to –0.022
Difference in LSM (SE) vs placebo*	1.175 (1.084)	–0.738 (1.040)	0.177 (0.793)	–	–
95% CI	–1.007 to 3.357	–2.829 to 1.353	–1.419 to 1.772	–	–

CI indicates confidence interval; LSM, least squares mean; SD, standard deviation; SE, standard error; UCEIS, Ulcerative Colitis Endoscopic Index of Severity.

*Comparison between treatments (each KHK4083 dose group versus placebo) was performed using an analysis of covariance model with change from baseline as the dependent variable and treatment group as the fixed effect, and baseline value as covariate.

TABLE 3: Total Mayo Score: Actual and Change from Baseline at Week 12 (Full Analysis Set)

	KHK4083 dose				
	**1 mg/kg (*n* = 7)**	**3 mg/kg (*n* = 8)**	**10 mg/kg (*n* = 22)**	**All doses (*n* = 37)**	**Placebo (*n* = 15)**
Baseline					
Mean (SD)	7.3 (1.5)	7.6 (1.1)	7.9 (1.5)	7.7 (1.4)	7.6 (0.9)
Week 12					
Mean (SD)	5.3 (2.9)	2.8 (1.0)	4.8 (2.8)	4.4 (2.6)	5.1 (2.8)
Change from baseline to week 12					
Mean (SD)	–2.0 (2.9)	–4.9 (1.8)	–3.1 (2.7)	–3.3 (2.7)	–2.5 (2.6)
95% CI	–4.7 to –0.7	–6.4 to –3.4	–4.3 to –1.9	–4.2 to –2.4	–4.0 to –1.1
LSM (SE)	–2.171 (0.969)	–4.896 (0.901)	–3.007 (0.546)	–	–2.566 (0.658)
95% CI of change	–4.121 to 0.222	–6.708 to –3.084	–4.105 to –1.909	–	–3.889 to –1.242
Difference in LSM (SE) versus placebo*	0.395 (1.169)	–2.331 (1.115)	–0.441 (0.856)	–	–
95% CI	–1.958 to 2.747	–4.574 to –0.087	–2.164 to 1.281	–	–

CI indicates confidence interval; LSM, least squares mean; SD, standard deviation; SE, standard error.
*Comparison between treatments (each KHK4083 dose group versus placebo) was performed using an analysis of covariance model with change from baseline as the dependent variable and treatment group as the fixed effect, and baseline value as covariate.

TABLE 4: Clinical Response, Clinical Remission, and Mucosal Healing Rates at Week 12 (Intent-to-Treat Population)

	KHK4083 dose				Placebo (n = 17)
	**1 mg/kg (n = 9)**	**3 mg/kg (n = 10)**	**10 mg/kg (n = 30)**	**All doses (n = 49)**	
Clinical response, n (%)	3 (33.3)	8 (80.0)	11 (36.7)	22 (44.9)	9 (52.9)
Clinical remission, n (%)	1 (11.1)	3 (30.0)	6 (20.0)	10 (20.4)	3 (17.6)
Mucosal healing, n (%)	1 (11.1)	3 (30.0)	8 (26.7)	12 (24.5)	4 (23.5)

TABLE 5: Endoscopic Healing Rates according to Total Mayo Score for Patients Who Have Both Assessments at Weeks 12 and 52

	**KHK4083 all doses combined (n = 27)**			**Placebo switched KHK4083 (n = 9)**		
	**Clinical response**	**Clinical remission**	**Mucosal healing**	**Clinical response**	**Clinical remission**	**Mucosal healing**
Week 12, n (%)	10 (37.0)	8 (29.6)	10 (37.0)	3 (33.3)	3 (33.3)	3 (33.3)
Week 52, n (%)	10 (37.0)	10 (37.0)	11 (40.7)	2 (22.0)	3 (33.3)	3 (33.3)

TABLE 6: Summary of KHK4083 Pharmacokinetic Parameters

Pharmacokinetic parameter	Mean (SD)		
	**KHK4083 1 mg/kg**	**KHK4083 3 mg/kg**	**KHK4083 10 mg/kg**
Week 0			
No. of patients	8	9	7
$AUC_{0-\tau}$, μg·day/mL	113 (31.4)	397 (64.9)	1066 (255)
$AUC_{0-\infty}$, μg·day/mL	166 (51.1)	680 (154)	1661 (486)
C_{max}, μg/mL	20.6 (6.21)	63 (5.87)	212 (52.5)
$t_{1/2}$, days	8.70 (2.47)	10.9 (3.02)	8.91 (3.87)
Week 10			
No. of patients	6	8	5
$AUC_{0-\tau}$, μg·day/mL	249 (91.2)	863 (178)	2092 (760)
C_{max}, μg/mL	31.8 (6.97)	108 (21.5)	291 (56.4)
$t_{1/2}$, days	11.7 (3.90)	14.6 (2.14)	9.01 (3.32)
Accumulation ratio	3.05 (2.30)	2.16 (0.214)	1.84 (0.683)
Week 48			
No. of patients	2	6	4
$AUC_{0-\tau}$, μg·day/mL	423 (115)	1469 (529)	3102 (856)
C_{max}, μg/mL	49.2 (29.6)	110 (24.5)	309 (81.6)
$t_{1/2}$, days	10.3 (2.89)	12.6 (3.79)	17.6 (4.45)

$AUC_{0-\tau}$ indicates area under the serum concentration-time curve over the dose interval; $AUC_{0-\infty}$, area under the serum concentration-time curve from time 0 to infinity; C_{max}, maximum serum concentration; SD, standard deviation; $t_{1/2}$, terminal elimination half-life.

FIGURES

FIGURE 1. Effect of KHK4083 on T-cell activation and memory cell expansion via OX40 blockade (reproduced and modified with permission from John Wiley & Sons Ltd. [Papp KA, et al. *J Eur Acad Dermatol Venereol.* 2017;31:1324–1332.]).

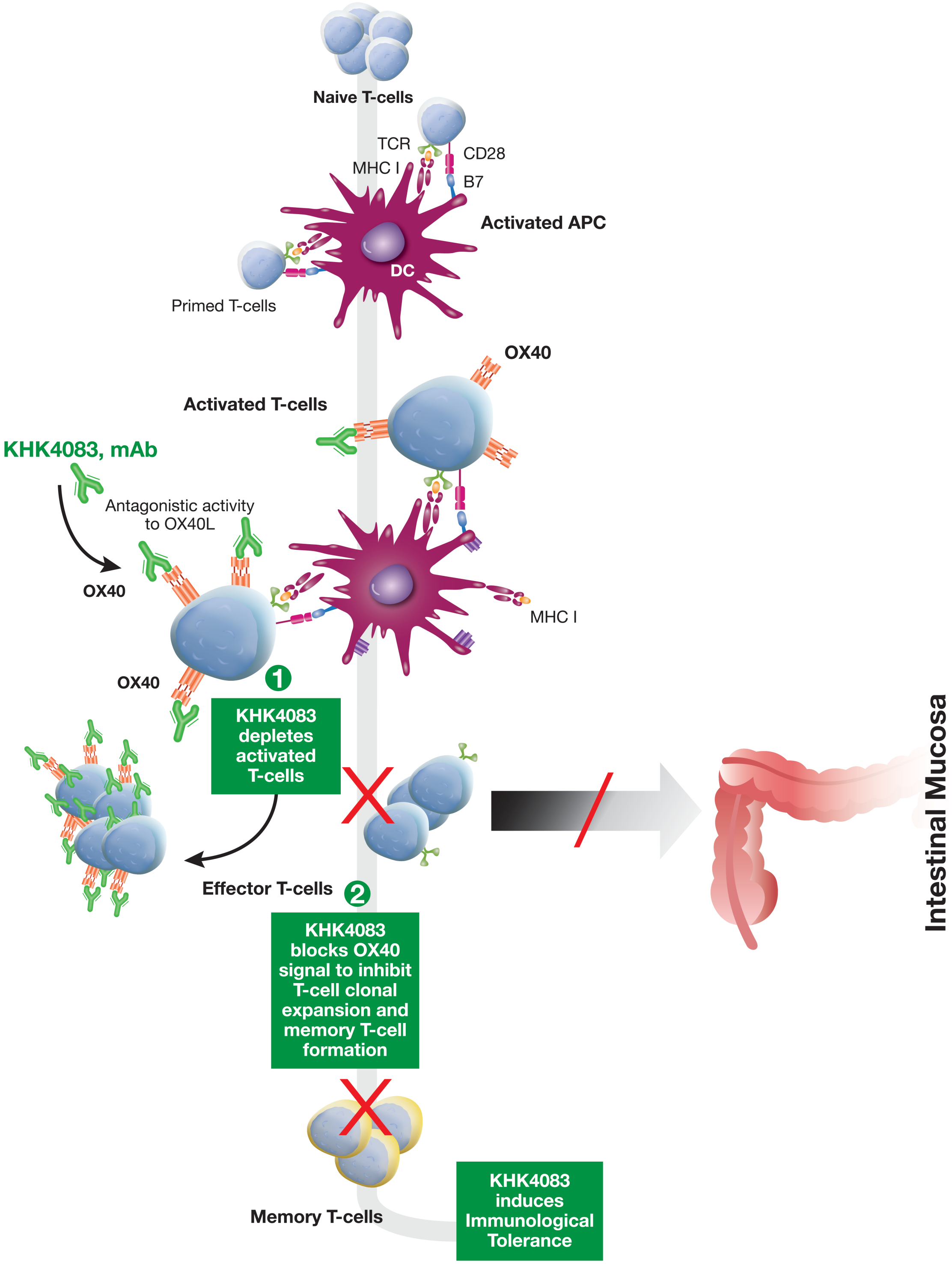

FIGURE 2. Representative image of OX40-stained cells (stained brown) against hematoxylin/eosin-stained background at baseline and at week 12 during remission after treatment with KHK4083.

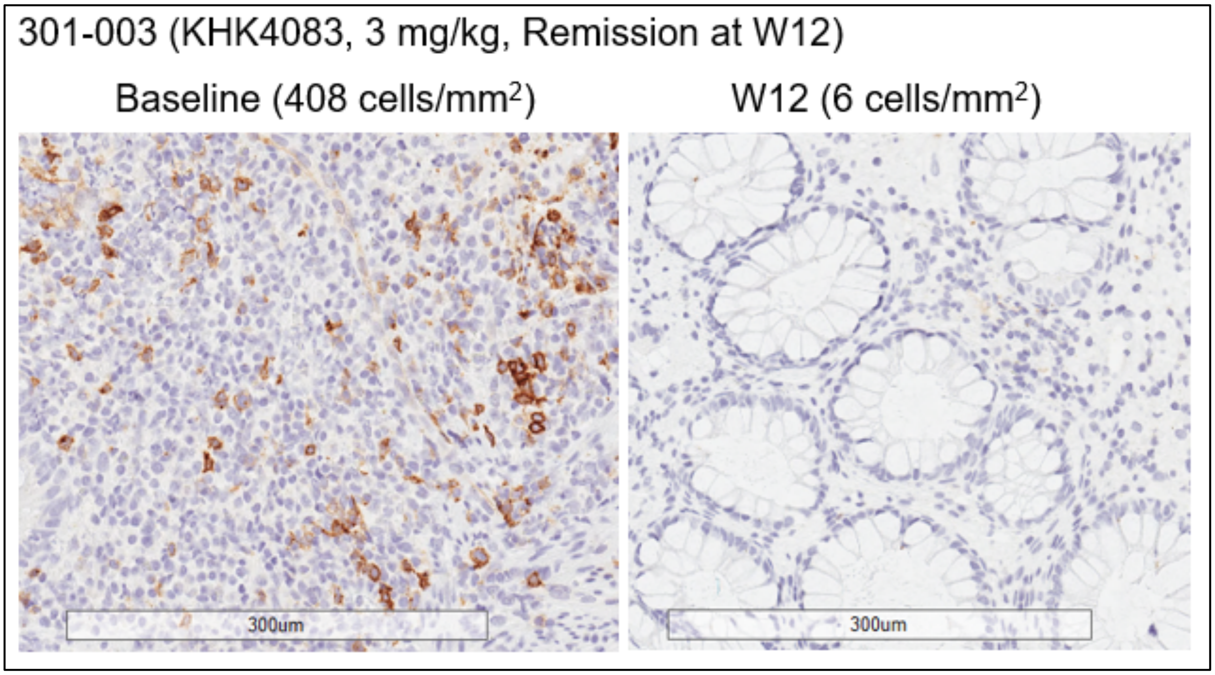

FIGURE 3. CONSORT diagram.

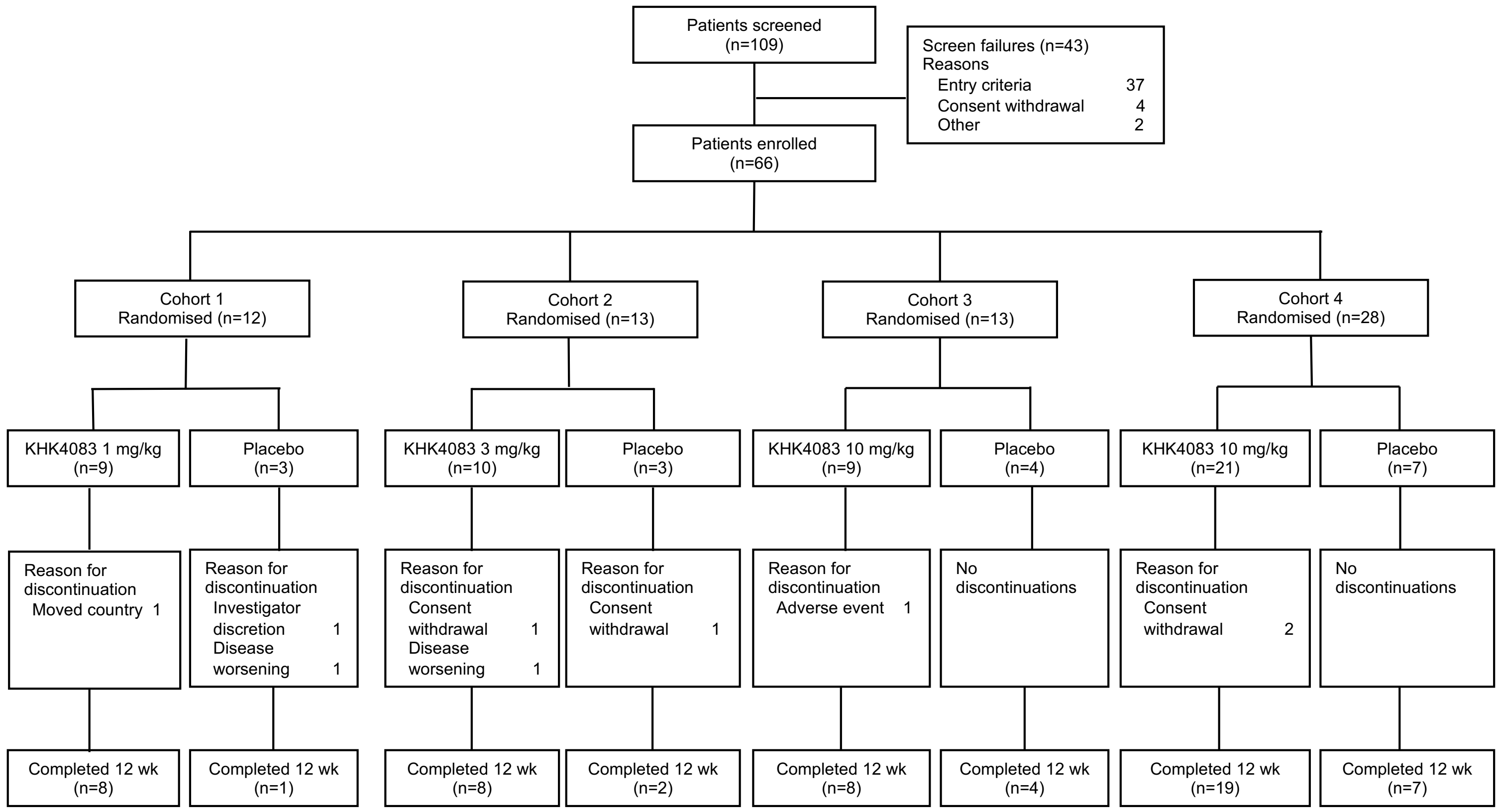

FIGURE 4. Correlation between baseline RHI score and clinical response at week 12. Each point represents the mean RHI score of multiple (up to 4) tissue biopsy specimens for each patient. Median baseline RHI score was 14.38 excluding patients who discontinued treatment by week 12: those above the median were designated as "more severe" and those below median as "less severe". Response rate was defined as the percentage of the patients who attained clinical response and/or remission. MH indicates mucosal healing; NR, no response; PBO, placebo; Rem, clinical remission; Res clinical response; RHI, Robarts Histological Index.

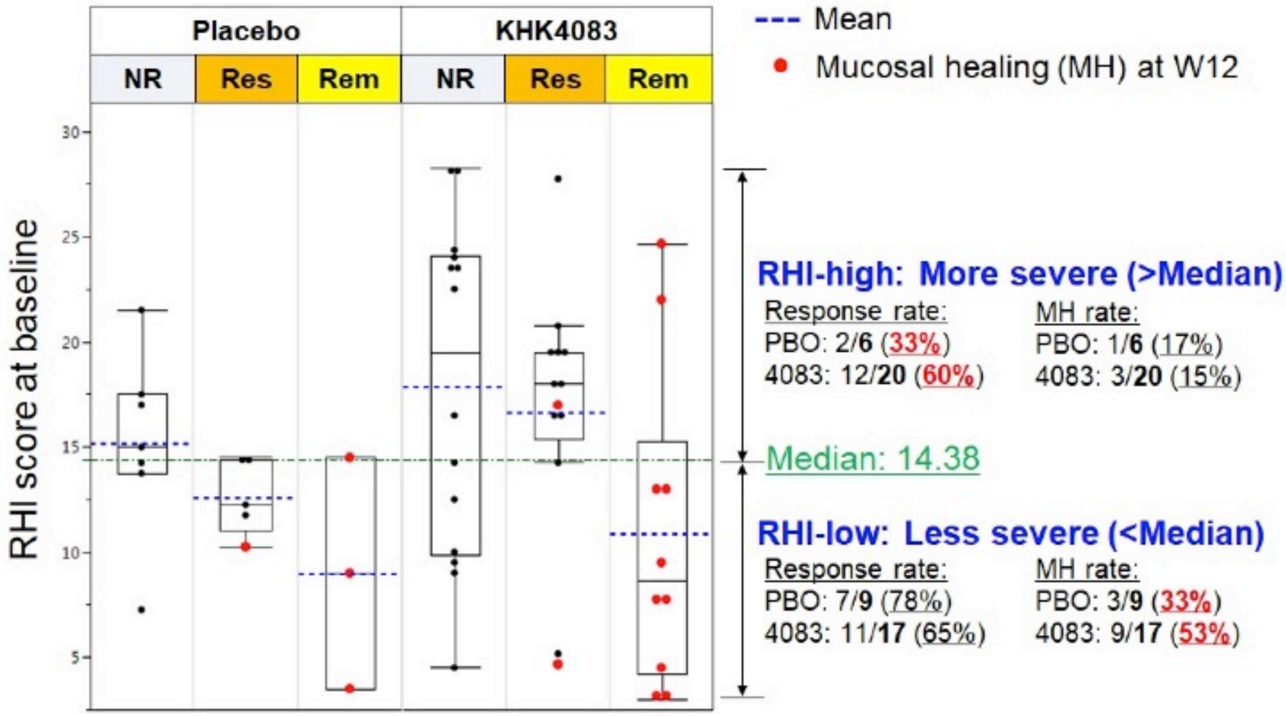

FIGURE 5. Mean (+ standard deviation) serum concentrations of KHK4083 following multiple intravenous administration (every 2 weeks for 12 weeks followed by every 4 weeks for 40 weeks) of KHK4083 at doses of 1, 3, and 10 mg/kg in patients with ulcerative colitis. Serum KHK4083 concentrations were measured over the dose interval for the doses administered at weeks 0, 10, and 48. Other data points show trough concentrations after each dose and/or terminal decline in concentrations after discontinuation of KHK4083 during the 12-week follow-up period.

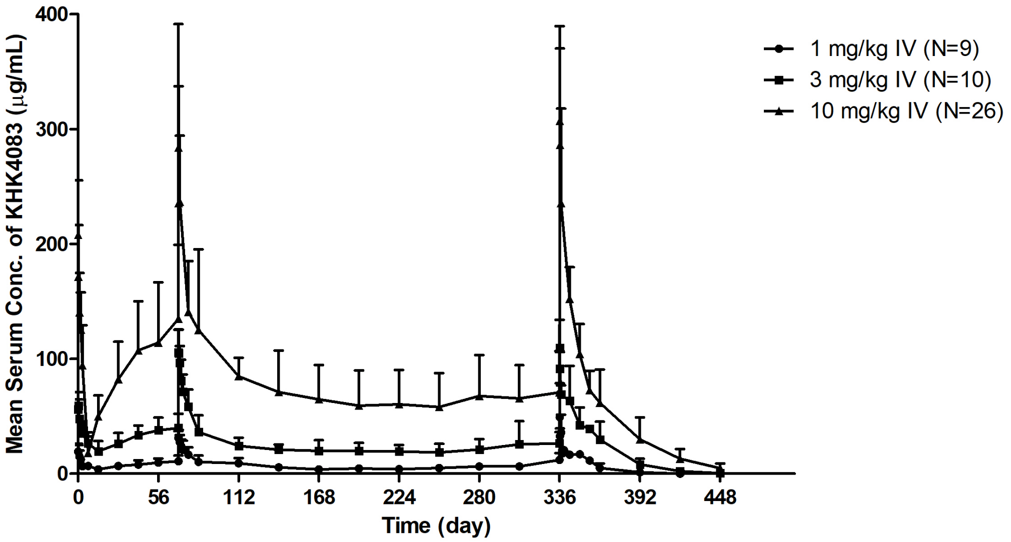

Supplement: otaa049_suppl_Supplementary_Material [file otaa049_suppl_supplementary_material.pdf]
